# Task cue influences on lexical decision performance and masked semantic priming effects: The role of cue-task compatibility

**DOI:** 10.3758/s13414-022-02568-2

**Published:** 2022-09-20

**Authors:** Alexander Berger, Wilfried Kunde, Markus Kiefer

**Affiliations:** 1 Department of Psychiatry, Section for Cognitive Electrophysiology, Ulm University, Leimgrubenweg 12, 89075 Ulm, Germany; 2 Department of Psychology, University of Würzburg, Würzburg, Germany

**Keywords:** Task cue, Task set, Semantic priming, Unconscious cognition, Task switching, Drift-diffusion models

## Abstract

Recent research demonstrated that mere presentation of a task cue influences subsequent unconscious semantic priming by attentional sensitization of related processing pathways. The direction of this influence depended on task-set dominance. Dominant task sets with a compatible cue-task mapping were supposed to be rapidly suppressed, while weak task sets showed more sustainable activation. Building on this research, we manipulated cue-task compatibility as instance of task-set dominance in two experiments and tested how masked semantic priming was influenced by actually performing the cued task (induction-task trials) or by mere cue presentation (task cue-only trials). In induction-task trials, the results of earlier research were replicated; semantic priming was larger following a semantic induction task compared to a perceptual induction task. In task cue-only trials, priming effects were reversed compared to induction-task trials in both experiments. Priming was larger for a perceptual compared to a semantic task set in task cue-only trials, indicating suppression of task sets following mere cue presentation in preparation for the upcoming lexical decision task. This notion of an inhibition of task sets after mere cue presentation was further supported by switching-related costs and changes of task-set implementation throughout the experiment. The absence of a moderator role of cue-task compatibility for task cue effects on priming in the present study suggests that the precise time course of task-set activation and inhibition in response to task cues as a function of cue-task compatibility might depend on specific experimental settings.

**Supplementary Information:**

The online version contains supplementary material available at 10.3758/s13414-022-02568-2.

## Introduction

Informing an individual about a task that has to be performed is often realized by presenting so-called task cues. Presenting such cues before stimulus presentation is supposed to activate the relevant task set in advance, thereby facilitating subsequent task performance (Meiran, 1996; Schuch & Koch, 2003). A task set can be defined as the configuration of the cognitive system required for task execution. It includes the relevant stimulus dimensions as well as the mapping of stimulus features to responses (Monsell, 2003; Rogers & Monsell, 1995). Linking a task cue to the respective task set can be established by instruction and practice (Rogers & Monsell, 1995). Facilitating effects of task cues on task performance were, for example, demonstrated in task-switching research, such that switching between different tasks is facilitated if a task cue is shown before stimulus presentation (Kiesel et al., 2010; Koch et al., 2010, 2018).

### Task set influences on masked semantic priming

Most relevant for the purpose of the present study, besides influences on performance in a task at hand, task sets also modulate unconscious processing in line with the attentional sensitization model of unconscious cognition (Kiefer & Martens, 2010). Previous research demonstrated this effect of task sets on masked semantic priming (Kiefer, 2019; Kiefer et al., 2019; Kiefer & Martens, 2010; Martens et al., 2011; Ulrich et al., 2014). Semantic priming, as employed in the paradigm by Kiefer and colleagues, is characterized by a faster and less error-prone response to a target word in a lexical decision task (LDT, word/pseudoword decision; e.g., Kiefer, 2002; Kiefer & Spitzer, 2000) if it is preceded by a semantically related prime word, indicating that semantic associations between the beforehand-presented prime word and the target word facilitate processing the target. Semantic priming effects also occur if the prime word is masked, i.e. if a mask, typically letter strings, is presented before and after the prime word (Kiefer, 2002). Masked primes are typically not consciously perceived (Breitmeyer, 2007), therefore rendering masked semantic priming within the LDT a possible tool to investigate unconscious semantic processing.

According to the attentional sensitization model (Kiefer & Martens, 2010), activated task sets sensitize corresponding processing pathways, thereby also influencing unconscious processing of a stimulus. Hence, an activated semantic task set should sensitize semantic processing pathways and facilitate semantic processing of a prime, whether it is visible or not, therefore enhancing masked semantic priming. In line with these predictions, semantic priming was shown to be larger after performing a semantic compared to a perceptual classification task, a so-called induction task (Kiefer, 2019; Kiefer et al., 2019; Kiefer & Martens, 2010; Martens et al., 2011; Ulrich et al., 2014), reflecting a larger functional overlap of the semantic processing of a prime word and the task set of the semantic induction task compared to the task set of the perceptual induction task. While it is difficult to determine the exact size of this functional overlap, it should certainly be greater for a semantic than for a perceptual task set. In line with this reasoning, results for different forms of unconscious priming reflected the respective differences in functional overlap of task sets and the respective priming process. For instance, masked visuo-motor priming (Martens et al., 2011) or pictorial evaluative priming (Kiefer et al., 2017) was larger following a perceptual than following a semantic induction task (for a comparison of influences of shape vs. color task sets on masked visuo-motor priming, see Zovko & Kiefer, 2013).

### Task cue influences on masked semantic priming

Based on previous research, which showed that masked and unmasked task cues can trigger task sets and influence subsequent performance (Lau & Passingham, 2007; Mattler, 2003; Reuss et al., 2011), Kiefer et al. (2019) investigated whether the above-mentioned modulation of masked semantic priming by semantic and perceptual induction tasks also occurs with the mere presentation of task cues. To test this hypothesis, Kiefer et al. (2019) conducted three experiments. They combined induction-task trials, i.e., before the LDT a semantic versus perceptual task set was induced by a semantic versus perceptual classification task with so-called task cue-only trials, i.e., a task cue was directly followed by the LDT, omitting the induction task on which the corresponding task set was to be applied. While in induction-task trials the result pattern of earlier studies was replicated, i.e., semantic priming was enhanced following a semantic compared to a perceptual induction task, the result pattern in task cue-only trials of Experiments 1 and 2 was reversed. Priming was larger following a perceptual compared to a semantic task cue. This pattern was supposed to reflect suppression of task sets in task cue-only trials (Kiefer et al., 2019). In task cue-only trials, where the LDT is immediately presented after the task cue, there is a conflict between the activated task set triggered by the task cue and the required task set for performing the LDT. Consequently, cued task sets supposedly have to be suppressed in order to perform the LDT, which results in inverted priming effects compared to induction-task trials, as suppression of task sets is associated with a de-sensitization of the related processing pathways (Kiefer et al., 2019; Kiefer & Martens, 2010).

### Task-set dominance and the time course of task-set activation

In a third experiment, task-set dominance was shown to moderate the priming pattern in task cue-only trials. Task-set dominance was previously manipulated by variations in stimulus-response mappings, i.e., in dominant conditions, stimuli and responses matched (e.g. Huestegge & Koch, 2013: auditory stimuli – vocal response; Jost et al., 2017: spatial stimuli – left/right response), while mappings of stimulus and responses were arbitrary or incompatible in the weak conditions. Dominant task sets were shown to receive more inhibition during task switching compared to weak task sets (Jost et al., 2017). In contrast to this previous work, which varied task-set dominance across different tasks within participants, Kiefer and colleagues varied task-set dominance between participants in the third experiment of this study: Task-set dominance was manipulated by varying the cue-task compatibility, i.e., “a priori associations between task cues and task elements” (Kiefer et al., 2019, p. 62). Color patches served as task cues. In version A, the so-called “dominant” version, the initial letter of the color of the patches matched the decision categories, i.e., a red (German: “**r**ot”) cue indicated the perceptual decision (round/elongated decision; German: “**r**und”), while a blue (German: “**b**lau”) cue indicated the semantic decision (living/non-living decision; German: “**b**elebt”). For version B, the “weak” version, these assignments were reversed.

As expected, the priming pattern was qualitatively different for dominant compared to weak task sets. In the dominant task-set condition (version A), priming was larger in perceptual task-cue only trials compared to semantic task cue-only trials, reflecting the pattern of Experiments 1 and 2 (where task cues were compatible with the response categories of the induction task). In the weak condition (version B), this pattern was reversed, i.e., priming was larger in semantic compared to perceptual task cue-only trials. Presumably, dominant task sets had to be inhibited rapidly and strongly in order to perform the LDT and are accordingly already suppressed when the masked prime is processed. In contrast, weak task sets with arbitrary cue-task mappings should take more time to be activated, as the mapping of cues and decision categories is less clear compared to compatible cue-task mappings, therefore requiring less inhibition compared to dominant task sets, when the absence of an induction task is evident. Accordingly, they are supposed to be still activated during processing of the masked prime, resulting in a sensitization of semantic processing of the prime following semantic task sets (Kiefer et al., 2019).

Furthermore, besides influences of task-set dominance, inhibition of task sets might also vary during the course of the experiment. For instance, differences between switch and repetition trials decreased if experiment duration was long enough to induce fatigue (Lorist et al., 2000). As activating a cued task set in the present study is associated with a task set switch (the end of a trial was always a LDT), one could assume that inhibition of task sets decreases throughout the experiment, because participants become less motivated to always activate the task set when it only has to be applied in half of the trials, i.e., in induction-task trials (see also the repetition bias in voluntary task switching, e.g., Arrington & Logan, 2004; Mayr & Bell, 2006; Mittelstädt et al., 2018). In an exploratory analysis, it is interesting to assess the temporal course of task-cue effects throughout the experimental session.

### Overview of the study

The present study aimed to further elucidate the role of task-set dominance for modulatory effects of task cues associated with semantic versus perceptual task sets on masked semantic priming. Previous work indicated that whether task sets were activated or suppressed after mere cue presentation depended on properties of the cues, i.e., cue-task compatibility, as outlined above. The present research extends this earlier work in three respects resulting in the following three major goals.

#### First goal

The moderating role of task-set dominance on task-set influences on priming was previously assessed only with non-verbal color cues (Kiefer et al., 2019). Hence, in two experiments, we aimed to manipulate cue-task compatibility at different strengths by more explicitly altering the cues and their relation to the cued task. In Experiment 1A of the present study, we realized this cue-task compatibility manipulation of task-set dominance with verbal letter cues. In the dominant condition, the task cue “R” indicated the perceptual decision (German: “**r**und”; English: “round”), while the task cue “B” indicated the semantic decision (German: “**b**elebt”; English: “living”). Hence, task cues and the first letter of the decision category were the same. These assignments were reversed in the weak condition. Furthermore, in Experiment 1B of the present study, we implemented a presumably more powerful manipulation of cue-task compatibility to test if the dominance effect depends on the strength of cue-task compatibility. For creating a dominant task-set condition, the verbal labels for the decision categories themselves (“rund”, engl. “round”; “belebt”, engl. “living”) were presented as task cues of the respective task set. With regard to the weak task-set condition, the arbitrary cues “$$$” and “§§§§§” indicated the perceptual and the semantic decision, respectively, counter-balanced across participants. With these arbitrary symbols, decision categories and cue properties lack any association, presumably resulting in even weaker task sets than in the weak condition in Experiment 1A.

#### Second goal

Previous work on task-cue effects on masked semantic priming were based on traditional analyses of reaction times (RTs) and error rates (ERs) (Kiefer et al., 2019). To further differentiate the processes modulated by task cues as well as induction tasks on masked semantic priming, we provide two additional levels of analyses. First, we employed estimations of drift-diffusion models. Drift-diffusion models (Ratcliff, 1978; Voss, Nagler, & Lerche, 2013a) use single-trial RT and ER data to map task performance to different parameters associated with cognitive processes. We focused on analyses of drift rates and non-decision times, as the drift rate reliably indexes semantic priming (Berger et al., 2021; Lerche & Voss, 2017; Voss, Rothermund, et al., 2013b), whereas the non-decision time is related to task-switching costs (Ging-Jehli & Ratcliff, 2020; Schmitz & Voss, 2012, 2014) as well as priming processes (Berger et al., 2021; Gomez et al., 2013). Drift-diffusion models possibly have the power to elaborate in more detail the processes triggered in task-cue only and induction-task trials and to permit disentangling influences on semantic priming from influences on general task performance. Second, we assessed the time course of brain activation via electroencephalography (EEG) as an additional tool to investigate priming effects in line with earlier studies using only induction-task trials (Kiefer & Martens, 2010; Martens et al., 2011). Semantic priming manifests in the modulation of the so-called N400 component (Kiefer, 2002; Kutas & Hillyard, 1980), a negative event-related potential (ERP) typically peaking around 400 ms (Kutas & Federmeier, 2010). The N400 in response to a target word preceded by a semantically related prime word is less negative compared to a target word preceded by an unrelated prime. Analyses of the N400 component may reveal how sensitizing and de-sensitizing semantic pathways affects semantic processing at a neural level.

#### Third goal

For exploratively investigating changes in task-set inhibition or activation throughout the experiment, we separated the trials of the experiment into early and late blocks and analyzed priming effects depending on block number. If it holds true that participants are less likely to always activate the task set after cue presentation with longer duration of the experiment, priming differences between task sets in task cue-only trials should decrease throughout the experiment.

#### Hypotheses

In line with earlier research, we expected stronger inhibition of dominant compared to weak task sets in task cue-only trials (Jost et al., 2017; Kiefer et al., 2019). Accordingly, we assumed priming in task cue-only trials to be larger for perceptual task sets in the dominant task-set condition, while priming should be larger for semantic task cue-only trials for weak task sets. Furthermore, we expected this pattern to be more pronounced in Experiment 1B compared to Experiment 1A due to a more effective dominance manipulation. For induction-task trials, we did not expect an influence of task-set dominance on the priming pattern (Kiefer et al., 2019). In induction tasks, task sets have to be applied, and task-set application should result in a comparable task-set activation in both dominance conditions. Consequently, we hypothesized semantic priming to be larger following a semantic induction task compared to a perceptual induction task in both dominance conditions of both experiments.

## Methods

### Experiment 1A

The experimental procedure, the planned analyses, the required sample size, as well as outlier and exclusion criteria were pre-registered at the Open Science Framework (https://osf.io/gahxy). Participants were naive concerning the real purpose of the experiment. Informed consent was obtained from all individual participants included in the study. The local ethics committee of Ulm University approved both experiments of the present study. The procedures used in this study adhere to the tenets of the Declaration of Helsinki.

#### Participants

We recruited a total number of 63 right-handed (Oldfield, 1971), native German-speaking participants. We excluded N = 12 participants from data analysis for the following reasons: an error in the experimental software (N = 1), above chance performance in the masked prime identification task (N = 6, see *Paradigm* section), high proportion of artifacts in the EEG signal (N = 2), mean RT in induction tasks or in the LDT exceeding ±2 SD of the sample mean (N = 3). The remaining sample consisted of N = 51 participants, with N = 26 participants assigned to the weak condition and N = 25 participants assigned to the dominant condition. Mean age was 23.07 years (SD = 3.34); 76.5% of the participants were female (N = 39).

#### Paradigm

Participants were randomly assigned to the two task-set dominance conditions. In the dominant task-set condition, task cues were the letter “R” for the perceptual decision (round/elongated decision, German: “rund”) and the letter “B” for the semantic decision (living/non-living decision, German “belebt”). A reversed assignment was used for the weak task-set condition, i.e., the letter “B” indicated the perceptual decision, while the letter “R” indicated the semantic decision.

Before the start of the main experiment, participants practiced the experimental procedure with a set of stimuli not overlapping with the stimulus set of the main experiment. First, they practiced the LDT as well as the induction tasks separately. Second, they practiced 30 trials with the same sequence as in the main experiment, consisting of 15 induction-task trials and 15 task cue-only trials. During the practice block, after each response, a string indicated whether the response was correct or incorrect. Participants were instructed to respond as quickly and as accurately as possible. They were not informed about the presence of the masked prime.

**Fig. 1:**
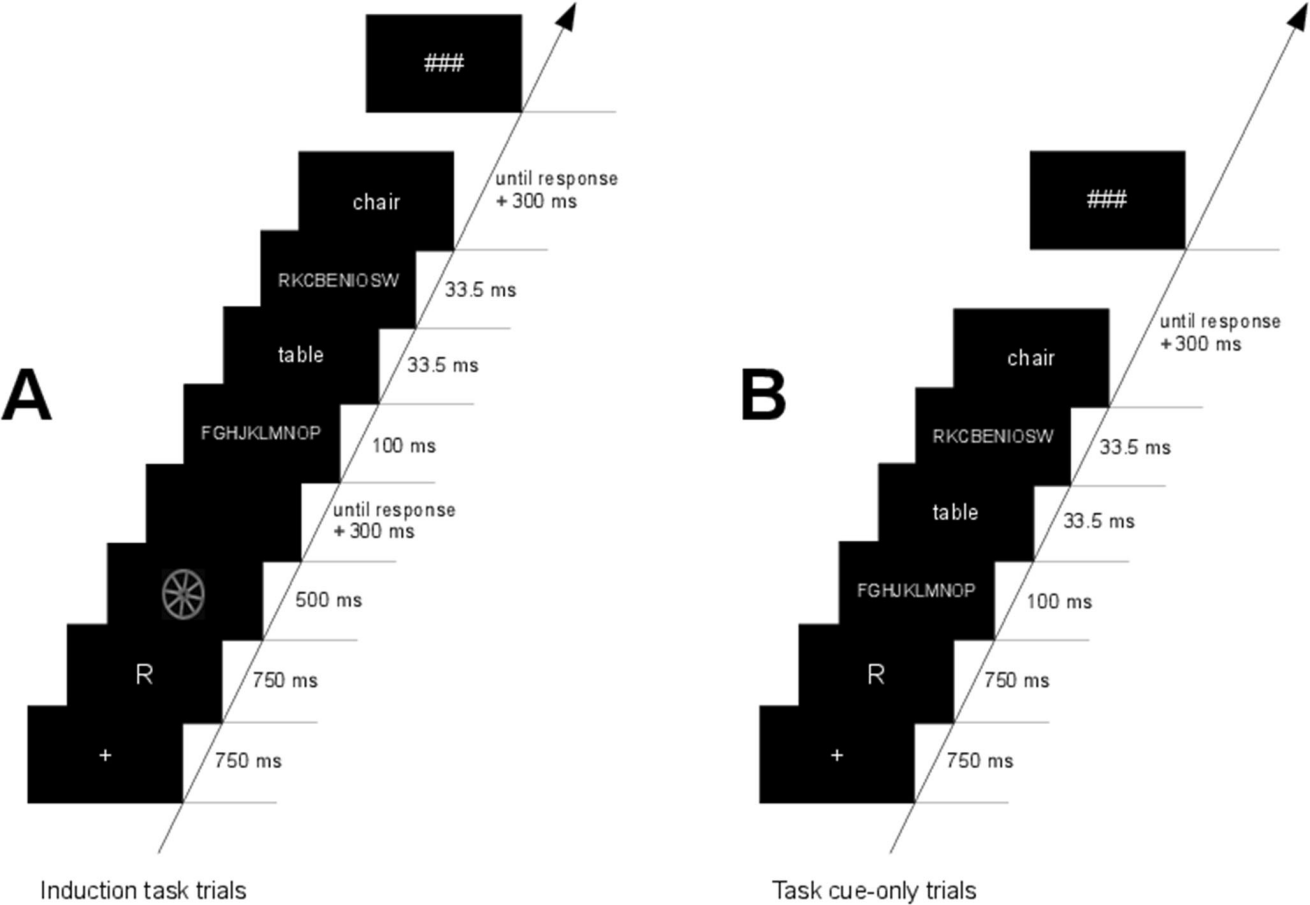
Sequence of induction-task trials (**panel a**) and task cue-only trials (**panel b**). Shown is an example sequence for Experiment 1A, in which letters served as task cues. In induction-task trials, the task cue indicates the decision that has to be made on the subsequently presented picture (round/elongated or living/non-living decision). Afterwards, the masked primed lexical decision task follows. In task-cue only trials, the lexical decision task immediately follows the task cue

The main experiment consisted of induction-task trials and task cue-only trials, which were intermixed and presented in random order. Accordingly, participants could not predict whether the next trial would be an induction task or a task-cue only trial. Figure 1 shows the sequence of events in induction-task trials and in task cue-only trials.

Both trial types started with a fixation cross for 750 ms,^1^ followed by a task cue for 750 ms, indicating the semantic or the perceptual decision. In induction-task trials, the task cue was followed by a picture for 500 ms, on which the semantic (living/non-living decision) or perceptual (round/elongated decision) task set had to be applied. A blank screen was shown until the participant responded plus an additional blank screen (300 ms) after the response. Afterwards, the LDT was presented. The LDT consisted of a forward mask, consisting of ten random capital letters (100 ms), the prime (33.5 ms), a backward mask consisting of ten random capital letters (33.5 ms), and the target, which was presented until a response was given. Participants had to decide whether the target was a meaningful German word or a pronounceable but meaningless pseudoword. At the end of each trial, after a 300-ms blank screen, three hash marks served as a break sign, indicating to the participants to start the next trial with a self-paced button press. In task cue-only trials, the LDT was immediately presented after the offset of the task cue with the same stimulation parameters as in induction-task trials. Participants responded by pressing buttons on a response box. The responses “living” and “round” in induction tasks as well as the “word” decision in the LDT were mapped on the index finger of the right hand. The responses “non-living,” “elongated,” and “pseudoword” were mapped on the middle finger of the right hand. The main experiment consisted of 640 trials. After a block of 80 trials, a screen indicated a break with a longer duration. The experiment included an equal number of induction-task trials and task cue-only trials (each 320 trials).

After the end of the main experiment, participants performed a prime identification task, assessing whether or not they show prime awareness. The prime identification task consisted of 80 trials. The sequence of a trial was identical to task-cue only trials, except that in half of the trials, a letter string consisting of a sequence of nine repeated identical capital letters replaced the prime word. The letter was randomly selected for each trial. The participants’ task was to discriminate between random letter strings and words. They were instructed to only focus on the sequence between the masks and to focus on accuracy. If they were not able to consciously perceive the masked words or letter strings, they were encouraged to guess. We defined prime awareness, which served as the exclusion criterion (see *Participants* section), as above-chance performance in the prime identification task. Participants were considered prime aware if they responded correctly in more than 61.25% of the trials. This proportion was calculated based on the upper 95% confidence interval of a binomial distribution with guessing probability p = 0.5.

The experimental stimulation was achieved using PsychoPy (Peirce et al., 2019), version v2020.2.4. Stimuli were presented on a cathode ray tube monitor (refresh rate 60 Hz) with white font against a black background synchronous with the screen refresh. Participants were seated 80 cm from the screen. The experimental procedure including practice, main experiment, and prime identification task lasted about 1.5 h. Including the preparation of the EEG, the total duration was about 3 h.

#### Material

Stimuli were taken from previous studies (Kiefer et al., 2019; Kiefer & Martens, 2010). Stimuli for the induction tasks were 320 grey-scaled pictures (each 170 × 216 pixels) depicting non-living and living objects. Half of these pictures (n = 160) were used for the perceptual induction task, depicting a round or elongated object (each n = 80). The other half (n = 160) depicted a living or non-living object (each n = 80) and were used for the semantic induction task. The distribution of the task-irrelevant decision category (shape for the semantic induction task and animatedness for the perceptual induction task) did not significantly differ between task sets, *χ*^*2*^(1) = 1.52, *p* = 0.218. The stimulus list for the LDT included 320 word-pseudoword pairs and 320 word-word pairs, i.e., half of the targets were meaningful German words, the other half were pseudowords. Primes were always meaningful words. Primes and targets were on average five letters long. Considering word-word pairs, half of the primes (n = 160) were semantically related to the target (e.g. “table” – “chair”), the other half were semantically unrelated to the target (e.g. “car” – “heaven”, n = 160). Stimuli were divided into four lists, which were assigned to the participants of both task-set dominance conditions in a counter-balanced fashion. These lists differed in the assignment of the LDT stimuli to a semantic/perceptual induction task or a semantic/perceptual task cue-only trial. Furthermore, we checked that pictures in induction tasks were always semantically unrelated to the prime and target in the LDT of the same trial.

#### Drift-diffusion models

Drift-diffusion models serve to analyze cognitive processes in two-choice decision tasks jointly using single-trial RT and ER data (Ratcliff, 1978; Voss, Nagler, & Lerche, 2013a). The model consists of four main parameters: the drift rate *ν*, the decision threshold *a*, the non-decision time *t0*, and the starting point *z*. It assumes an information accumulation process until one of two thresholds is hit and the response is initiated. The decision threshold *a* represents the amount of information needed to separate both thresholds. The drift rate *ν* represents the speed of information accumulation, and the starting point *z* the position between both thresholds, where the information accumulation process (i.e., drift) starts. Response execution processes as well as stimulus encoding processes are mapped to the non-decision time *t0*. Considering the present analyses, we mapped correct and incorrect responses to both the thresholds and fixed the starting point *z* to *a*/2 (for a similar approach, see Berger et al., 2021).

We were interested in influences on semantic processing, which should be reflected by drift rates (Berger et al., 2021; Lerche & Voss, 2017; Voss, Rothermund, et al., 2013b), as well as influences on switching-related processes indexed by non-decision times (Ging-Jehli & Ratcliff, 2020; Schmitz & Voss, 2012, 2014). Accordingly, we analyzed the model parameters *ν* and *t0* depending on trial type, task set, semantic relatedness, and task-set dominance, while the decision threshold *a* was fixed across conditions for the sake of model parsimony. Drift rates and non-decision times were estimated varying for trial type (induction-task trial vs. task cue-only trial), task set (semantic vs. perceptual), and semantic relatedness (related vs. unrelated) in separate models for the dominant and weak condition of the between-subject factor task-set dominance. We calculated separate models for the different participants of the dominant and weak conditions to reduce the number of factors per model. Estimated parameters of the dominant and weak condition were concatenated for statistical analysis. Drift-diffusion models were estimated in a hierarchical Bayesian framework using the tool “HDDM,” version 0.6.0 (Wiecki et al., 2013).

Model convergence was assessed by evaluating the Monte Carlo error statistic, by visual inspection of the chains, and by means of the Gelman-Rubin statistic (see Berger et al., 2021; Wiecki et al., 2013). Each criterion indicated that the models for both dominance conditions converged.

#### EEG recording and ERP extraction

Recording and processing of the EEG was similar to earlier studies of our research group (e.g. Harpaintner et al., 2020; Popp et al., 2016). The EEG was recorded at 64 equidistant sintered Ag/AgCl electrodes, placed into an electrode cap (EasyCap, Herrsching, Germany). The reference electrode was placed between FCz and Cz, the grounding electrode between AFz and Fz. Infra- and supra-orbital electrodes as well as an electrode between the medial canthi were used to monitor eye movements. The signal was continuously recorded at a sampling rate of 500 Hz using a BrainAmp amplifier (BrainProducts, Gilching, Germany). EEG data were processed using BrainVision Analyzer software (version 2.2; BrainProducts, Gilching, Germany). Impedances were kept below 5 kΩ. The data were filtered using a high-pass (0.1 Hz, 12 dB/oct), a low-pass (30 Hz, 24 dB/oct), and a Notch filter (50 Hz). Motor and alpha artifacts were excluded by visual inspection. Ocular artifacts were removed using Independent Component Analysis. Channels with a noisy signal were replaced by the average of four surrounding channels using Hjorth Nearest Neighbors interpolation. Additionally, further artifacts were automatically excluded using the following criteria: maximal allowed voltage step 50 μV/ms, maximal allowed difference of values in a segment 100 μV, maximal amplitude 70 μV, minimal amplitude -70 μV.

Data were segmented -467 ms to 1,000 ms relative to onset of the LDT target and baseline corrected for the time window between -367 ms and -167 ms, i.e., before the masked prime interval, to ensure that there was no change in visual stimulation during the baseline interval. Artifact-free segments with a correct response were averaged and re-referenced to the average reference. Subjects with any condition consisting of less than 20 segments after preprocessing were excluded from data analysis (compare *Participants* section).

By visual inspection, we identified the peak of the N400 component to fall within the time window between 450 ms and 550 ms after target onset. Mean activity in this time window was extracted as the index of the N400 component. We extracted the N400 component at electrodes P1, P2, PO1, PO2, PO3, and PO4 in line with earlier studies (Kiefer, 2002; Kiefer et al., 2017; Kiefer & Brendel, 2006; Kiefer & Martens, 2010; Kiefer & Spitzer, 2000).

Based on our hypothesis of increased inhibition of dominant task sets, we analyzed oscillatory brain activity in the theta frequency range as also mentioned in the preregistration. Theta activity was shown to be related to cognitive control processes (Cavanagh & Frank, 2014). These analyses did not reveal any differences between the task-set dominance conditions. Consequently, we refrain from reporting these results, as they do not add further insights into the role of task-set dominance beyond the behavioral and N400 results.

### Experiment 1B

Experiment 1B was pre-registered at the Open Science Framework (https://osf.io/ahk35). The design of Experiment 1B differed from Experiment 1A mainly in terms of the task-set dominance manipulation. Therefore, we will only describe the deviations of Experiment 1B from Experiment 1A.

#### Participants

A total number of 52 right-handed, native German-speaking participants were recruited for Experiment 1B. Four participants were excluded due to mean RT in induction tasks or in the LDT exceeding ±2 SD of the sample mean (N = 2), a high proportion of artifacts in the EEG signal (N = 1), and above-chance performance in the masked prime identification task (N = 1). From the remaining N = 48 participants, half were assigned to the dominant and the other half to the weak task-set dominance condition (each N = 24). Participants in the remaining sample were on average 22.54 years old (SD = 3.06). N = 30 of the participants were female (62.5%).

#### Paradigm and material

Participants were randomly assigned to the dominant and weak task-set dominance conditions. In the dominant condition, the task cue “rund” (English: “round”) indicated the perceptual decision, while the task cue “belebt” (English: “living”) indicated the semantic decision. In the weak condition, the task cues “$$$” and “§§§§§” served as indicators of the semantic and perceptual decision, respectively, counter-balanced across participants. The material and paradigm of the main experiment was otherwise identical to Experiment 1A.

In the prime identification task, we changed the number of the letter strings. Accordingly, a letter string, which had to be discriminated from prime words, consisted in Experiment 1B of a repetition of seven identical capital letters. This adjustment was based on the observation of participants in Experiment 1A numerically showing objective prime awareness without subjectively reporting any awareness when debriefed afterwards. In the prime identification tasks of Experiment 1A, prime words were on average five letters long (range three to seven), while letter strings were all nine letters long, and masks consisted of ten letters. Hence, to be able to confine above-chance performance in the prime identification task to awareness of the prime content and not to differences in the visual stimulation, we restricted letter strings in Experiment 1B to seven letters.

#### Drift-diffusion models and processing of EEG data

Estimation of drift-diffusion models was the same as for Experiment 1A. Just as for Experiment 1A, each assessed convergence criterion (Monte Carlo error statistic, visual inspection of the chains, Gelman-Rubin statistic) indicated convergence of the drift-diffusion models for both task-set dominance conditions. Processing of EEG data was the same as for Experiment 1A.

### Analyses for Experiments 1A and 1B

To foreshadow the results, the result patterns of Experiments 1A and 1B were quite similar. While both experiments were pre-registered and planned separately, we decided to collapse data of both experiments for statistical analysis to enhance power and to shorten manuscript length.^2^ Nevertheless, to account for possible differences between experiments, we included experiment as an additional between-participants factor in the respective analyses. When a particular effect was moderated by experiment, we calculated the respective analyses separately for each experiment (see Online Supplementary Material (OSM)).

#### Analysis of the prime identification task

We calculated *d’* sensitivity measures (Green & Swets, 1966) to quantify visibility of the masked primes among letter string distractors in the prime identification task. Hits were defined as correct responses to masked prime words, while false alarms were defined as incorrect responses to masked letter strings. We applied a correction for extreme proportions (Hautus, 1995) with the help of the “R” package “psycho” (Makowski, 2018), to account for response strategies like always pressing the same button. We tested if *d’* measures significantly deviated from zero using a two-tailed one-sample t-test. In order to assess if possible awareness of primes affected the magnitude of priming, we correlated *d’* measures and RT priming scores (mean RT difference semantically unrelated – semantically related conditions) as well as *d’* measures and ER priming scores (mean ER difference semantically unrelated – semantically related conditions). Correlations were tested against zero using a two-sided t-test.

#### Statistical analyses

Behavioral data was preprocessed using “R” (R Core Team, 2020), plots were created with help of the R package “ggplot2” (Wickham, 2016). Frequentist inferential statistics were calculated with “JASP” (JASP Team, 2020; Love et al., 2019), while Bayesian inferential statistics were calculated using R with the help of the packages “BayesFactor” (Morey & Rouder, 2021) and “bayestestR” (Makowski et al., 2019). Preprocessing of data for RT, ER, and drift-diffusion model analyses included exclusion of trials with a RT exceeding ±2 SD of the individual mean RT per participant (cf. Berger & Kiefer, 2021). For the RT analyses, incorrect responses were excluded as well. We calculated repeated-measures ANOVAs for mean RTs and mean ERs in induction tasks, for mean RTs and mean ERs in the LDT as well as for the mean amplitude of the N400 component. Considering the drift-diffusion model parameters *ν* and *t0*, we calculated Bayesian repeated-measures ANOVAs to be consistent with the statistical framework in which these parameters were estimated. For evaluating the evidence for an effect indexed by the Bayes factor (BF) in the Bayesian ANOVA analyses, we used the recommended values by Wagenmakers et al. (2017). Note that we omit reporting linear mixed model analyses mentioned in the pre-registration. We calculated these analyses, which showed comparable results to the ANOVA analyses. We only report results of the ANOVAs for the sake of brevity and consistency with the reported analyses of previous studies on task cue influences. To further describe the relevant interactions, we report the effect size Cohen’s *d* for the respective priming effects, using the following formula: ${d}_{priming}=\left({mean}_{unrelated}-{mean}_{related}\right)/\left(\frac{SD_{unrelated}+{SD}_{related}}{2}\right)$. Accordingly, for the frequentist as well as the Bayesian ANOVAs, the same measure is used to further quantify the interactions for the sake of consistency.

For RTs and ERs in induction tasks, we calculated a 2 × 2 × 2 repeated-measures ANOVA with the within-subjects factor task set and the between-subjects factors task-set dominance and experiment. For RTs, ERs, and drift-diffusion models parameters of the LDT, we calculated 2 × 2 × 2 × 2 × 2 repeated-measures ANOVAs with the within-subjects factors trial type (induction-task trials vs. task cue-only trials), task set (semantic vs. perceptual), semantic relatedness (related vs. unrelated), and the between-subjects factors task-set dominance (dominant vs. weak) and Experiment (1A vs. 1B). Considering the analysis of the N400 component, the respective ANOVA additionally included the within-subjects factor brain hemisphere (left vs. right).

#### Changes of task-set activation throughout the experiment

We analyzed whether the activation of task sets changed throughout the experiment by adding the factor block into the analyses.^3^ These analyses were not pre-registered and accordingly have to be considered exploratory. All available trials were split into three blocks based on their temporal sequence in the experiment. To be precise, all trials in each experimental cell (all possible combinations of the factors semantic relatedness, trial type, and task set for each subject) were sorted according to when they were presented throughout the experiment. Afterwards, these trials were split into three equally large blocks.^4^ We compared RTs and ERs of the first and third block, as possible changes of task-set activation should be most pronounced when comparing the beginning and the end of the experiment. Hence, we calculated for mean RTs and mean ERs in induction tasks 2 × 2 × 2 × 2 repeated-measures ANOVAs (within-subjects factors: task set, block (first vs. third); between-subjects factors: task-set dominance, experiment). For mean RTs and mean ERs in the LDT, we accordingly calculated 2 × 2 × 2 × 2 × 2 × 2 repeated-measures ANOVAs (within-subjects factors: task set, trial type, semantic relatedness, block (first vs. third); between-subjects factors: task-set dominance, experiment). We also tried to estimate drift-diffusion models including the factor block, but model evaluation indicated failed convergence for some conditions, likely a consequence of the reduced number of available trials per experimental cell to 13 (40 / 3 ≈ 13). Hence, the N400 components could not be analyzed as well depending on the factor block, as an available number of 13 trials per experimental cell was lower than the above-defined threshold of a minimum of 20 trials for conducting EEG analyses. Therefore, only analyses of RTs and ERs are reported for investigating changes of task-set activation throughout the experiment, and all these analyses should be interpreted with caution.

## Results

### Prime identification task and outlier exclusion

For Experiment 1A, the mean of *d’* scores was 0.176 (*SD* = 0.297). A two-tailed one-sample t-test revealed a significant deviation from zero for *d’* measures, *t*(50) = 4.24, *p* < 0.001, *95% CI*: 0.093–0.260. The correlation of *d’* and RT priming scores, *t*(49) = 0.01, *p* ≈ 1, *95% CI*: -0.275–0.276, as well as the correlation of *d’* and ER priming scores, *t*(49) = -0.98, *p* = 0.332, *95% CI*: -0.399–0.142, did not significantly deviate from zero. For Experiment 1B, the mean of *d’* scores was 0.150 (*SD* = 0.307). The *d’* measures significantly differed from zero, *t*(47) = 3.38, *p* = 0.001, *95% CI*: 0.060–0.239. The correlation of *d’* measures and RT priming scores, *t*(46) = -0.26, *p* = 0.795, *95% CI*: -0.319–0.248, as well as the correlation of *d’* measures and ER priming scores, *t*(46) = -1.01, *p* = 0.318, *95% CI*: -0.414–0.143, did not significantly differ from zero. In induction tasks of Experiment 1A, on average 3.9% of RTs were excluded as outliers (range 0–6.9%), while in Experiment 1B 3.8% were excluded (range 0–7.8%). For the LDT, in Experiment 1A the number of on average excluded RTs was 3.9% (range 1.7–6.2%), while it was 4.0% (range 1.0–6.4%) in Experiment 1B.

### Induction tasks

There was a significant effect of task set on RTs, *F*(1, 95) = 14.72, *p* < 0.001, ${\eta}_p^2$ = 0.134, indicating faster responses in perceptual compared to semantic induction tasks. The interaction of task set and task-set dominance reached significance as well, *F*(1, 95) = 8.51, *p* = 0.004,${\eta}_p^2$ = 0.082; differences between task sets were especially pronounced for dominant task sets: RTs for perceptual compared to semantic task sets were about 32 ms faster for dominant and 5 ms faster for weak task sets, respectively. No remaining effect reached significance, all *F*s < 2.39, all *p*s > 0.125. For ERs, there was no significant effect, all *F*s < 1.55, all *p*s > 0.216.

### Conventional analysis of the behavioral data of the lexical decision task

**Fig. 2:**
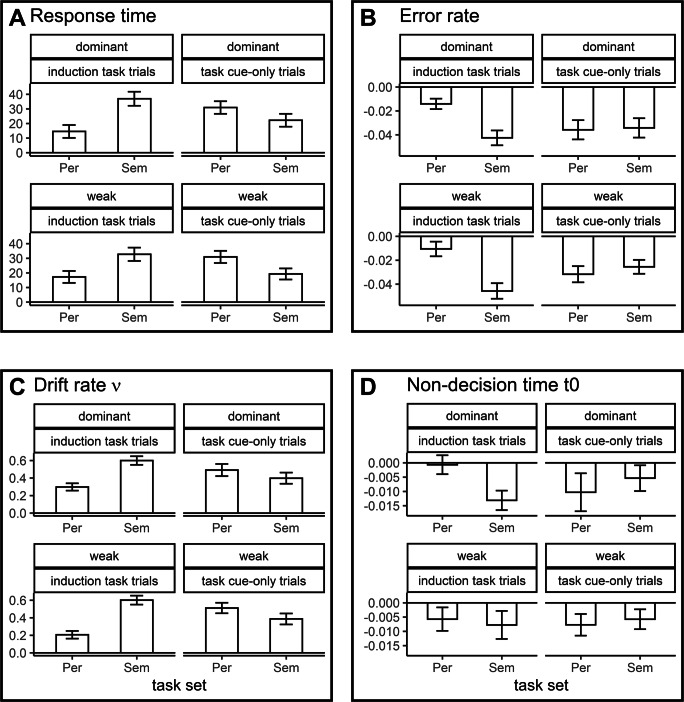
Priming scores for response times (**panel a**), error rates (**panel b**), drift rates (**panel c**), and non-decision times (**panel d**) for the lexical decision task collapsed across Experiments 1A and 1B. Larger absolute values indicate larger priming. The whiskers show standard errors. The unit for response times is the millisecond, for error rates proportion correct responses. The units for the drift-diffusion model parameters are arbitrary. “Per” indicates the perceptual, “Sem” the semantic task set

For the descriptive statistics of RTs and ERs in the LDT in Experiments 1A and 1B, see Tables 1 and 2, respectively. Considering the analysis of RTs, there was a main effect of trial type, *F*(1, 95) = 14.14, *p* < 0.001, ${\eta}_p^2$ = 0.130, indicating faster responses in induction-task trials compared to task cue-only trials. The main effect of semantic relatedness was significant as well, *F*(1, 95) = 233.56, *p* < 0.001, ${\eta}_p^2$ = 0.711, reflecting the typical priming effect with faster responses for related compared to unrelated prime-target pairs. Neither the main effect of task set, task-set dominance nor experiment reached significance, all *F*s < 2.97, all *p*s > 0.087. However, on a descriptive level, RTs were on average faster in the dominant compared to the weak task-set condition (about 24 ms). Furthermore, RTs were about 24 ms faster in Experiment 1A compared to Experiment 1B. The three-way interaction task set × trial type × semantic relatedness was significant, *F*(1, 95) = 24.57, *p* < 0.001, ${\eta}_p^2$ = 0.205. This effect reflected a reversed priming pattern following semantic compared to perceptual task sets in induction-task trials versus task cue-only trials. As can be seen in Fig. 2, panel A, in induction-task trials, priming was larger following a semantic (*d*_*priming*_ = 0.450) than a perceptual task set (*d*_*priming*_ = 0.204). In task cue-only trials, the reversed pattern was observed: priming was larger following a perceptual (*d*_*priming*_ = 0.415) compared to a semantic task set (*d*_*priming*_ = 0.276). This pattern was moderated by task-set dominance and experiment, indicated by the respective five-way interaction task set × trial type × semantic relatedness × task-set dominance × experiment, *F*(1, 95) = 6.76, *p* = 0.011, ${\eta}_p^2$ = 0.066. For Experiment 1A, the reversed priming pattern depending on trial type and task set was more pronounced in the weak task-set condition, while it was more pronounced in the dominant task-set condition in Experiment 1B. However, the direction of the modulation was the same for both weak and dominant task sets for both experiments (for separate analyses per experiment, see the OSM). Furthermore, the interaction task set × trial type reached significance, *F*(1, 95) = 6.12, *p* = 0.015, ${\eta}_p^2$ = 0.060. While average RTs for perceptual and semantic task sets were nearly equal for induction-task trials (difference about 0.3 ms), RTs for semantic task sets were slightly faster than for perceptual task sets in task cue-only trials (about 6 ms). No remaining interaction reached significance, all *F*s < 2.75, all *p*s > 0.100.

**Table 1:** Descriptive statistics of the behavioral measures and drift-diffusion model parameters for the lexical decision task in Experiment 1A

Task set dominance	Task set	Trial type	Semantic relatedness	RT	ER	*ν*	*t0*
dominant	semantic	induction-task trials	related	564.7 (63.6)	1.8 (2.3)	3.28 (0.43)	0.37 (0.03)
			unrelated	591.3 (67.4)	6.0 (4.7)	2.75 (0.30)	0.39 (0.03)
		task cue-only trials	related	583.0 (69.5)	4.2 (4.0)	3.13 (0.34)	0.40 (0.04)
			unrelated	609.2 (75.7)	7.0 (6.0)	2.78 (0.45)	0.40 (0.04)
	perceptual	induction-task trials	related	566.0 (64.8)	2.8 (3.2)	3.23 (0.31)	0.38 (0.03)
			unrelated	586.2 (81.2)	3.5 (3.3)	2.92 (0.37)	0.38 (0.03)
		task cue-only trials	related	582.0 (63.6)	4.3 (4.1)	3.10 (0.44)	0.39 (0.04)
			unrelated	611.4 (71.2)	7.4 (5.3)	2.64 (0.38)	0.40 (0.03)
weak	semantic	induction-task trials	related	593.4 (70.7)	1.9 (3.3)	3.05 (0.46)	0.38 (0.05)
			unrelated	627.5 (80.0)	6.2 (5.8)	2.52 (0.39)	0.39 (0.04)
		task cue-only trials	related	609.8 (81.5)	2.9 (3.3)	3.30 (0.52)	0.41 (0.04)
			unrelated	624.8 (77.8)	4.8 (4.4)	2.96 (0.37)	0.41 (0.04)
	perceptual	induction-task trials	related	603.3 (76.2)	3.9 (3.4)	2.87 (0.33)	0.38 (0.04)
			unrelated	619.5 (79.3)	3.9 (3.4)	2.76 (0.39)	0.39 (0.04)
		task cue-only trials	related	607.3 (69.8)	3.8 (4.1)	3.15 (0.46)	0.40 (0.04)
			unrelated	640.1 (76.3)	5.3 (4.5)	2.81 (0.46)	0.42 (0.05)

Values are given as mean and SD (in parentheses). The unit for the response times (RTs) is ms, for the error rate (ER) percent incorrect. The units for the drift-diffusion model parameters drift rate (*ν*) and non-decision time (*t0*) are arbitrary

**Table 2:** Descriptive statistics of the behavioral measures and drift-diffusion model parameters for the lexical decision task in Experiment 1B

Task set dominance	Task set	Trial type	Semantic relatedness	RT	ER	*ν*	*t0*
dominant	semantic	induction-task trials	Related	584.5 (65.6)	2.1 (2.8)	3.30 (0.39)	0.39 (0.03)
			Unrelated	632.3 (91.4)	6.5 (4.7)	2.63 (0.34)	0.40 (0.03)
		task cue-only trials	Related	610.1 (71.2)	2.4 (3.4)	3.16 (0.34)	0.41 (0.04)
			Unrelated	628.2 (77.9)	6.5 (4.6)	2.71 (0.35)	0.41 (0.03)
	perceptual	induction-task trials	Related	600.8 (75.0)	1.9 (2.5)	3.12 (0.37)	0.39 (0.03)
			Unrelated	609.6 (75.5)	4.1 (3.2)	2.84 (0.30)	0.39 (0.03)
		task cue-only trials	Related	613.1 (76.9)	3.8 (3.6)	2.86 (0.47)	0.39 (0.06)
			Unrelated	645.7 (78.6)	7.8 (6.2)	2.34 (0.30)	0.40 (0.05)
weak	semantic	induction-task trials	Related	617.2 (82.9)	1.2 (1.8)	3.23 (0.44)	0.40 (0.04)
			Unrelated	648.6 (83.8)	6.1 (4.9)	2.56 (0.46)	0.40 (0.04)
		task cue-only trials	Related	622.5 (72.8)	2.7 (2.9)	3.33 (0.53)	0.42 (0.04)
			Unrelated	646.3 (73.8)	5.9 (5.6)	2.89 (0.46)	0.43 (0.04)
	perceptual	induction-task trials	Related	624.8 (75.9)	1.8 (2.1)	3.00 (0.46)	0.39 (0.05)
			Unrelated	643.1 (81.3)	4.0 (4.9)	2.69 (0.48)	0.40 (0.04)
		task cue-only trials	Related	627.4 (76.6)	1.8 (2.1)	3.42 (0.48)	0.43 (0.05)
			Unrelated	656.3 (77.4)	6.8 (5.7)	2.73 (0.48)	0.43 (0.05)

Values are given as mean and SD (in parentheses). The unit for the response times (RTs) is ms, for the error rate (ER) percent incorrect. The units for the drift-diffusion model parameters drift rate (*ν*) and non-decision time (*t0*) are arbitrary

For ERs, there was a main effect of trial type, *F*(1, 95) = 18.04, *p* < 0.001, ${\eta}_p^2$ = 0.160, reflecting more errors in task cue-only trials compared to induction-task trials. The effect of semantic relatedness, *F*(1, 95) = 144.08, *p* < 0.001, ${\eta}_p^2$ = 0.603, reflected less errors in related prime-target pairs compared to unrelated prime-target pairs. Neither the main effect of task set, task-set dominance nor experiment was significant, all *F*s < 1.19, all *p*s > 0.278. The two-way interactions trial type × task-set dominance, *F*(1, 95) = 4.37, *p* = 0.039, ${\eta}_p^2$ = 0.044, trial type × task set, *F*(1, 95) = 12.85, *p* < 0.001, ${\eta}_p^2$ = 0.119, task set × semantic relatedness, *F*(1, 95) = 7.15, *p* = 0.009, ${\eta}_p^2$ = 0.070 and semantic relatedness × experiment, *F*(1, 95) = 8.20, *p* = 0.005, ${\eta}_p^2$ = 0.079, reached significance. For dominant task sets as well as for perceptual task sets, the effect of trial type (i.e., more errors in task cue-only trials) was more pronounced. Collapsed across trial types, the effect of semantic relatedness was more pronounced for semantic task sets. Furthermore, ER priming was larger in Experiment 1B. Comparable to the analysis of RTs, there was a significant three-way interaction task set × trial type × semantic relatedness, *F*(1, 95) = 17.31, *p* < 0.001, ${\eta}_p^2$ = 0.154. Figure 2, panel b shows in induction-task trials a more pronounced priming effect following semantic (*d*_*priming*_ = -1.164) compared to perceptual task sets (*d*_*priming*_ = -0.368), while for task cue-only trials the priming effect was slightly larger for perceptual task sets (semantic: *d*_*priming*_ = -0.692, perceptual: *d*_*priming*_ = -0.744). No remaining interaction reached significance, all *F*s < 1.81, all *p*s > 0.181.

### Drift-diffusion model analyses of the behavioral data of the lexical decision task

Tables 1 and 2 show the descriptive statistics for the parameters of the drift-diffusion model analysis, respectively, for Experiment 1A and Experiment 1B. For the analysis of the drift rate *ν*, there was extreme evidence for an effect of semantic relatedness, *BF* = 4.53*e^70^, with larger drift rates for related prime-target pairs. Considering the main effect of task set, there was strong evidence, *BF* = 11.36, reflecting overall larger drift rates for semantic task sets in the LDT. This effect was additionally moderated by trial type, reflected by the interaction task set × trial type, *BF* = 193.98. In task cue-only trials, drift rates were on average larger for semantic compared to perceptual task sets, while in induction-task trials no pronounced differences between task sets were observed. Furthermore, there was extreme evidence for the interaction trial type × task-set dominance, *BF* = 5.72*e^18^: for dominant task sets, drift rates were on average larger in induction-task trials, while for weak task sets, mean drift rates were larger in task cue-only trials. Strong evidence was found for the interaction semantic relatedness × experiment, *BF* = 14.90, reflecting a larger priming effect in Experiment 1B. Considering the theoretical relevant three-way interaction trial type × task set × semantic relatedness, there was extreme evidence, *BF* = 3.45*e^5^. As can be seen in Fig. 2, panel c, for induction-task trials, priming on drift rate *ν* was larger following semantic (*d*_*priming*_ = 1.477) compared to perceptual task sets (*d*_*priming*_ = 0.643). For task cue-only trials, the reversed pattern was observed; priming was larger following perceptual (*d*_*priming*_ = 1.068) than following semantic task sets (*d*_*priming*_ = 0.912). All remaining main effects and interactions showed at most moderate evidence, all *BF*s < 5.94.

Considering the non-decision time *t0*, there was extreme evidence for the main effects for semantic relatedness (extreme evidence: *BF* = 190.62) and trial type (extreme evidence: *BF* = 4.18*e^22^). Non-decision times were smaller for related compared to unrelated prime-target pairs and non-decision times were larger for task cue-only trials in comparison to induction-task trials. Furthermore, there was extreme evidence for the interaction trial type × task-set dominance, *BF* = 383.90. The effect of trial type was more pronounced for weak task sets. There was no considerable evidence for any other effect, all *BF*s < 1.41.

### Analysis of the N400 ERP component

Descriptive statistics of the N400 component are shown in Supplementary Table C1 and Supplementary Table C2 (OSM), respectively, for Experiments 1A and 1B. Crucial for our hypotheses of a different modulation of semantic priming by trial type and task set depending on task-set dominance were only effects involving the factor semantic relatedness, as only these effects reflect modulating influences on semantic processing indexed by the N400 component. Hence, we only report effects involving the factor semantic relatedness. The main effect of semantic relatedness was significant, *F*(1, 95) = 102.87, *p* < 0.001, ${\eta}_p^2$ = 0.520. ERPs were more positive for related compared to unrelated prime-target. Furthermore, the interaction semantic relatedness × brain hemisphere, *F*(1, 95) = 18.90, *p* < 0.001, ${\eta}_p^2$ = 0.166, reached significance. The priming effect was larger over the left hemisphere. No remaining effect involving the factor semantic relatedness reached significance, all *F*s < 3.40, all *p*s > 0.067.

### Changes in task-set activation throughout the experiment

For analyzing changes in task-set activation during the course of the experimental session, we will only report significant effects involving the experimental factor block, as only these effects add additional information to the analyses reported above. Considering RTs in induction tasks, there was a significant main effect of block, *F*(1, 95) = 103.10, *p* < 0.001, ${\eta}_p^2$ = 0.520, indicating faster responses in the third block compared to the first block. This effect was moderated by task-set dominance, *F*(1, 95) = 10.61, *p* = 0.002, ${\eta}_p^2$ = 0.100. RT differences between task-set dominance conditions became smaller throughout the experiment. Furthermore, the four-way interaction task set × task-set dominance × block × experiment reached significance as well, *F*(1, 95) = 5.24, *p* = 0.024, ${\eta}_p^2$ = 0.052. RTs were consistently lower or comparable for perceptual than semantic task sets despite for the first block for weak task sets in Experiment 1B (see OSM). All remaining effects including the factor block did not reach significance, all *F*s < 2.91, all *p*s > 0.091. For the analysis of ERs in induction tasks, no effect involving the factor block was significant, all *F*s < 3.29, all *p*s > 0.072.

**Fig. 3:**
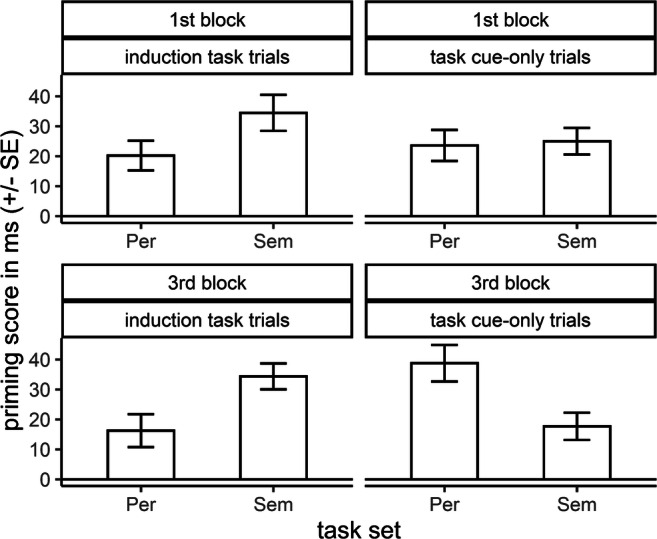
Priming scores for response times in the lexical decision task depending on block number in millisecond. Priming scores were collapsed across experiments and task-set dominance conditions. Larger values indicate larger priming. The whiskers show standard errors. “Per” indicates the perceptual, “Sem” the semantic task set

Analyses of RTs in the LDT (for descriptive statistics, see Table 3) revealed a significant main effect of block, *F*(1, 95) = 64.28, *p* < 0.001, ${\eta}_p^2$ = 0.404, reflecting faster RTs at the end of the experiment (third block) compared to the beginning of the experiment (first block). The interaction task set × trial type × block was significant as well, *F*(1, 95) = 6.30, *p* = 0.014, ${\eta}_p^2$ = 0.062, reflecting pronounced RT differences between task sets to be only present for the third block in task cue-only trials. Furthermore, and of particular theoretical relevance, the interaction of task set, trial type, and semantic relatedness was moderated by block on a descriptive level (see Fig. 3). In induction-task trials, priming was larger after semantic task sets (first block: *d*_*priming*_ = 0.362, third block: *d*_*priming*_ = 0.456) compared to perceptual task sets (first block: *d*_*priming*_ = 0.231, third block: *d*_*priming*_ = 0.195), both in the first and the third block (but being more pronounced in the third block). In contrast, for the first block in task cue-only trials, priming was quite comparable across task sets (semantic: *d*_*priming*_ = 0.295, perceptual: *d*_*priming*_ = 0.278), while for the third block, priming after mere cue presentation was larger after perceptual compared to semantic task sets (semantic: *d*_*priming*_ = 0.235, perceptual: *d*_*priming*_ = 0.489). However, the respective four-way interaction task set × trial type × semantic relatedness × block, *F*(1, 95) = 3.18, *p* = 0.078, ${\eta}_p^2$ = 0.032, as well as all remaining interactions including the factor block, all *F*s < 2.27, all *p*s > 0.134, did not reach significance. Considering the analyses of ERs in the LDT, only the interaction of semantic relatedness × task-set dominance × block was significant, *F*(1, 95) = 6.57, *p* = 0.012, ${\eta}_p^2$ = 0.065. For dominant task sets, average ER priming decreased throughout the experiment, while it increased for weak task sets. No remaining effect including the factor block reached significance, all *F*s < 2.49, all *p*s > 0.117.

**Table 3:** Descriptive statistics of the behavioral measures for the lexical decision task depending on block number collapsed across Experiments 1A and 1B

Task set dominance	Task set	Trial type	Semantic relatedness	RT		ER	
				1st block	3rd block	1st block	3rd block
dominant	semantic	induction-task trials	Related	587.3 (85.9)	568.2 (63.3)	1.4 (4.0)	2.9 (4.4)
			unrelated	629.3 (98.2)	602.8 (82.1)	6.0 (6.2)	6.0 (7.7)
		task cue-only trials	related	617.5 (72.8)	586.1 (79.1)	3.2 (5.6)	3.2 (4.8)
			unrelated	641.3 (90.0)	600.0 (66.3)	7.2 (8.1)	6.0 (7.9)
	perceptual	induction-task trials	related	595.9 (74.5)	575.0 (81.6)	2.0 (3.7)	3.4 (5.6)
			unrelated	617.5 (92.1)	589.5 (86.5)	4.0 (6.0)	5.1 (6.6)
		task cue-only trials	related	619.2 (82.6)	583.7 (72.8)	4.8 (6.4)	3.4 (5.2)
			unrelated	639.4 (84.1)	620.4 (86.8)	8.2 (9.2)	7.5 (8.0)
weak	semantic	induction-task trials	related	624.9 (98.7)	593.9 (72.0)	2.0 (4.6)	2.1 (2.8)
			unrelated	652.0 (94.4)	628.0 (80.9)	6.0 (8.0)	7.2 (8.1)
		task cue-only trials	related	635.6 (86.3)	599.3 (76.2)	4.2 (6.7)	1.2 (2.9)
			unrelated	661.9 (89.2)	620.6 (78.3)	5.2 (6.6)	6.5 (7.2)
	perceptual	induction-task trials	related	631.7 (82.7)	596.0 (83.5)	2.5 (4.3)	2.3 (4.4)
			unrelated	650.6 (95.7)	614.0 (79.5)	3.8 (5.6)	5.1 (7.9)
		task cue-only trials	related	631.1 (86.9)	604.0 (71.6)	2.8 (4.7)	3.1 (5.9)
			unrelated	658.0 (86.8)	644.8 (84.1)	4.6 (6.9)	7.0 (7.7)

Values are given as mean and SD (in parentheses). Data were collapsed across experiments, as experiment did not moderate the effect of block number on lexical decision task performance

## Discussion

In the present work, we investigated attentional influences of task cues associated with a semantic or a perceptual task set on subsequent masked semantic priming in so-called task cue-only trials. Previous work (Kiefer et al., 2019) indicated semantic priming to be modulated by the mere presentation of task cues, while the direction of this modulation depended on cue-task compatibility as an instance of task-set dominance. Based on these results, we investigated if the findings of Kiefer and colleagues extend to a different cue modality, i.e., verbal letter cues (Experiment 1A), and if the supposedly more effective cue-task compatibility manipulation in Experiment 1B compared to Experiment 1A influences task set inhibition and hence the priming pattern in task cue-only trials. Furthermore, on top of conventional analyses of RTs and ERs, we provided analyses of drift-diffusion models and analyses of the N400 ERP component to better separate attentional sensitization influences on masked priming from influences on general task performance. Note that we observed reliable priming effects in all analyses (RTs and ERs, drift rate and non-decision time in drift-diffusion models and N400 component). Therefore this study permits examining task set influences on priming at different levels of cognitive processes indexed by the different variables.

Contrary to our expectations, the cue-task compatibility manipulation did not modulate the priming pattern in task cue-only trials compared to induction-task trials. Furthermore, the hypothesized strength of this dominance manipulation did not play a modulatory role since there was a similar result pattern in Experiments 1A and 1B. In task cue-only trials, priming was consistently larger following a perceptual compared to a semantic task set. In induction-task trials, the pattern of earlier studies was replicated (Kiefer, 2019; Kiefer et al., 2019; Martens et al., 2011; Ulrich et al., 2014): priming was larger following a semantic compared to a perceptual induction task.

### General differences in task performance

Although task-set dominance did not influence the (de-)sensitization of semantic prime processing in task cue-only trials as indexed by the missing moderation of priming, dominance did affect general task performance in line with earlier research (Jost et al., 2017). Concerning induction tasks, RT differences between semantic and perceptual induction tasks were larger in the dominant condition. Activating and applying the appropriate task set may have been more demanding in the condition with weak cue-task mappings, therefore attenuating performance differences between the semantic and perceptual induction tasks. However, it should be mentioned that performance differences in induction tasks cannot account for the modulation of priming in the subsequent LDT. If modulation of priming is the consequence of differences in difficulty between semantic and perceptual induction tasks, priming should be the same for both task sets in task cue-only trials. Furthermore, the modulation of priming in induction-task trials should be remarkably smaller for the weak task-set condition, in which performance differences for induction tasks were less pronounced. However, the result pattern is inconsistent with such a simple explanation based on task difficulty.

Additional effects of task-set dominance were found on the drift rate in the LDT, which represents the speed of information accumulation (Ratcliff & McKoon, 2008; Voss, Nagler, & Lerche, 2013a). On average, drift rates were larger in induction-task trials compared to task cue-only trials for dominant task sets, while for weak task sets the opposite was observed. Correctly applying the task set in induction tasks presumably had been more difficult for incompatible than for compatible cue-task mappings, therefore increasing cognitive demands for the subsequent LDT in induction-task trials due to carry-over effects. Hence, in the weaker task-set condition, processing speed in the LDT in induction-task trials had been reduced in comparison to task cue-only trials. In contrast, for dominant task sets including a compatible cue-task mapping, the relative ease of applying the task set in induction tasks might have resulted in little carry-over effects on the LDT as reflected by the high drift rates, while the high demands of suppressing the highly activated, but irrelevant, cued task set in task cue-only trials (see the next section) lowered the drift rate in the LDT.

General differences between Experiments 1A and 1B, i.e., possible influences of the different cue-task compatibility manipulations, were largely absent. Neither were influences of task-set dominance particularly pronounced in one experiment, nor the modulation of priming. However, ER and drift rate priming effects were somewhat larger in Experiment 1B (see OSM), which nevertheless did not affect the modulation of priming by task sets depending on trial type.

#### Task-set inhibition in task cue-only trials

Four main findings regarding task performance can be seen as an additional index for an inhibition of task sets in task cue-only trials besides the priming modulation. First, responses were slower and more error prone in task cue-only trials. This finding was further supported by the drift-diffusion model analyses, showing larger non-decision times in task cue-only trials. Non-decision times were shown to reflect switching costs (Ging-Jehli & Ratcliff, 2020; Klauer et al., 2007; Schmitz & Voss, 2012, 2014). Accordingly, larger non-decision times in task cue-only trials could indicate an increased effort to get rid of the cued task set compared to induction-task trials, in which the cued task set had to be applied. However, this finding is confounded by differences in stimulation between induction-task trials and task cue-only trials, i.e., an additional blank screen after the response to the induction task was presented, where participants could prepare for the LDT, while the LDT immediately followed the task cue in task cue-only trials. Please also note that a direct comparison of LDT performance in induction task and task cue-only trials is difficult because these trial types also differ with respect to many other aspects: In induction-task trials, there is an additional stimulus, there is an additional response, and the onset of the LDT depends on the RT in the induction task.

Second, besides sequence effects within one trial, i.e., the sequences task cue – induction task – LDT and task cue – LDT, sequence effects between trials, for example semantic task cue-only trial – semantic induction-task trial, could indicate task set inhibition (cf. *n-2 inhibition costs*, e.g., Koch et al., 2010). We therefore analyzed trial-type repetition and task-set repetition effects on the performance in induction tasks (see OSM), as task-set application only occurred in induction tasks. To shortly summarize the findings, independent of task-set dominance, RTs in induction tasks were slowed if the preceding trial was a task cue-only trial with the same task set. This could presumably reflect costs associated with re-activating a previous (i.e., in the preceding task cue-only trial) suppressed task set.

Third, as induction-task trials and task cue-only trials occurred with the same probability, participants could not predict if an induction task follows a task cue. Consequently, if participants were less likely to prepare for a possible occurring induction task throughout the experiment (Lorist et al., 2000), i.e., were less likely to activate the corresponding task set in advance, they had at least to remember the cue. Otherwise, they would have to guess the required task set for the induction task, which should result in near-chance performance. However, if participants refrained from activating the task set in advance with longer experimental duration and moved on to only remembering the cue, RTs in induction tasks should increase, as facilitatory effects of in advance activated task sets on induction task performance would diminish. Contrary to these expectations, RTs in induction tasks decreased from the first to the third block, more likely representing a practice effect, i.e., application of pre-activated task sets is facilitated throughout the experiment.

Last, taking up the former line of reasoning, priming in task cue-only trials depended on block number on a descriptive level (*p* = 0.078). In induction-task trials, priming was larger for semantic compared to perceptual task sets in both blocks, while being slightly more pronounced in the third block in line with the overall performance facilitation in induction tasks during the course of the experiment. In task cue-only trials, in contrast, the priming pattern changed from the first to the third block: In the first block, priming was comparable across task sets, while priming in the third block was larger in perceptual compared to semantic task cue-only trials, indicating a differential modulation of priming by the task cues mainly at the end of the experiment. Suppression of the cued task set probably occurred only when task-set activation in task cue-only trials was strong enough to provide a sufficient conflict with the task set of the upcoming LDT. Conflicting task sets are the primary reason for task-set inhibition (Koch et al., 2010), and the amount of conflict supposedly increased during the course of the experiment.

To sum up, effects on general task performance, effects of task set repetition, changes in induction task performance, and the temporal course of the priming effects throughout the experiment render an inhibition of task sets in task cue-only trials a likely explanation of the reversed priming pattern in comparison to induction-task trials. Possible reasons why task-set dominance did not modulate the priming pattern in the LDT and how the inhibition of task sets is in line with the suggested time course of task set de-sensitization according to the attentional sensitization model are discussed in the following sections.

#### Influences of task cues on semantic priming

To resume our hypotheses, we expected different masked priming patterns in task cue-only trials depending on task-set dominance: For dominant task sets, priming was supposed to be larger following a perceptual task cue compared to a semantic cue, while for weak task sets the opposite pattern was expected. Although there were subtle differences in the magnitude of priming between task-set dominance conditions, the priming modulation by task sets and trial type was qualitatively the same for both weak and dominant task sets in both experiments.^5^

As outlined above, the inverse priming pattern in task cue-only trials compared to induction-task trials suggests task sets are suppressed in the LDT in task cue-only trials. According to the attentional sensitization model, if a task set is suppressed, the respective processing pathway should be de-sensitized as well (Kiefer & Martens, 2010). Hence, elevated priming in task cue-only trials for perceptual compared to semantic task sets indicated that a de-sensitization of task-irrelevant (concerning semantic priming) perceptual processing pathways resulted in a net increase of priming compared to when the task-relevant semantic task set was de-sensitized. In the present paradigm, the modulation of priming is therefore limited to the comparison of semantic and perceptual task sets. To evaluate the absolute amount of (de-)sensitization for semantic and perceptual task sets, a “neutral” task-set condition would be required, by including, for example, a simple response task as an additional induction task condition to index a baseline level of priming in the sample.

The modulation of priming was comparable for RTs, ERs, and drift rates. As drift diffusion model analyses specifically revealed an effect on drift rates, the present results indicate that attentional sensitization influences on masked semantic priming mainly concern the processing stage by enhancing versus attenuating prime-related advantages in processing the target (supposedly semantic pre-activation, see Berger et al., 2021). Accordingly, drift-diffusion model analyses, which separate responses into two main components, i.e. processing the stimulus (*ν*) and a non-decisional component (*t0*), revealed a different sensitivity of these components to the experimental manipulation. Drift rates reflected influences of task sets, which sensitized or de-sensitized the respective processing pathway depending on being activated in induction-task trials or being suppressed in task cue-only trials, resulting in increased or attenuated priming, while non-decisional processes (*t0*) reflected more general influences of the trial type.

However, the precise time course of the de-sensitization of task sets in task cue-only trials cannot be clearly identified according to the results of the present study. In order to be able to influence masked semantic priming, suppression of task sets needs to occur in time intervals of ongoing prime processing. As a result, suppression can also occur in time intervals after prime presentation – for instance, after target onset when semantic information of the prime and target is integrated. Task-switching research indicated that inhibition effects were largest at long intervals between cue and target (1,000 ms vs. 200 ms; Scheil & Kleinsorge, 2014). Accordingly, task sets might be suppressed only after onset of the target. In line with that reasoning, note that ERP-masked priming effects (N400) typically do not occur before about 400 ms after target onset (Deacon et al., 2000; Kiefer, 2002; Kiefer & Spitzer, 2000). It therefore suffices that the task set is suppressed in an interval until about 400 ms after target onset, i.e., until about 567 ms after the cue, to influence masked priming. Furthermore, task sets must not necessarily be fully suppressed to explain the observed priming modulation in task cue-only trials, which only requires semantic pathways to be relatively more de-sensitized after a semantic task cue compared to a perceptual one.

#### Absence of a moderator role of task-set dominance and a modulation of N400 priming

Nevertheless, it still remains unclear why a different result pattern with regard to the modulatory role of task-set dominance on task cue effects on priming was observed in comparison to Experiment 3 of Kiefer et al. (2019). First, it should be noted that the sample size of the present study was determined to achieve a power *β* = 0.9 for the replication of the four-way interaction in Experiment 3 of Kiefer and colleagues (see the pre-registration templates). Accordingly, as the power was sufficient, differences in the design likely are the only factor that could explain the deviating results between the studies. The experiments only differed in the modality of the task cues; Kiefer and colleagues (Experiment 3) used color cues, while task cues were verbal or symbolic in the present study. Experiments 1 and 2 of Kiefer et al. (2019), which only realized a compatible cue-task mapping, showed the same priming pattern as the present study, using verbal cues as well. Possibly, linking the task cue to the respective task requirements is qualitatively different for different cue modalities. For instance, recent work indicated the cue type to influence backward inhibition in task switching (cf. Gade & Koch, 2014; Houghton et al., 2009). Nevertheless, the weak condition of Experiment 1B in the present study indicated task-set inhibition in task cue-only trials even using a completely arbitrary symbolic cue-task mapping. Other differences between the present study and Kiefer et al. (2019) concerned the overall response speed of participants. In the present study, in order to conduct drift-diffusion model analyses, instruction of participants, and training before the main experiment emphasized response speed somehow stronger compared to the earlier study to achieve a more homogenous RT distribution. Consequently, responses were about 15–40 ms faster compared to the study of Kiefer and colleagues, which could influence the sensitive timing of processes throughout a trial in the complex paradigm combining induction task and task cue-only trials. Further studies could systematically investigate the influence of general response speed and test different instances of manipulating task-set dominance than cue-task compatibility.

Considering attentional sensitization influences on priming, we also assessed priming on a neural level, using the electrophysiological index of the N400 component. While N400 priming in general was reliable, we did not observe a modulation of the N400 by task sets and trial type. In the present study, effects on the behavioral measures and effects on the N400 therefore were dissociated, indicating N400 and behavioral measures do not reflect identical semantic processes. Accordingly, earlier results showing a different modulation of N400 priming in induction-task trials (Kiefer & Martens, 2010; Martens et al., 2011) could not be replicated. Earlier studies presented the different induction tasks (semantic vs. perceptual) block-wise, while they were shown randomly and intermixed with task cue-only trials in the present study. Possibly, ERPs show a different sensitivity to such a complex paradigm compared to RTs and ERs, resulting in large variability between trials concealing attentional sensitization influences.

## Conclusion

To sum up, in line with earlier work, the mere presentation of a task cue differentially influenced masked semantic priming compared to performing an induction task. In induction-task trials, semantic priming was enhanced following a semantic induction task and attenuated following a perceptual induction task. As a novel aspect of this study, drift diffusion modelling indicated the modulatory influence of task sets on priming to affect the drift rate, thereby specifying the locus of the effect, i.e., task sets influence the information accumulation process. In contrast, task cue-only trials increased semantic priming for perceptual task sets and attenuated priming for semantic task sets, for both dominant and weak task sets, using compatible and incompatible cue-task mappings, respectively. This modulation of priming indicated task sets to be suppressed during semantic processing in the LDT in task cue-only trials. Furthermore, as further novel aspects of this study, switching-related costs and changes of task-set implementation during the course of the experiment supported this notion of an inhibition of task sets after mere cue presentation. Manipulating cue-task compatibility with verbal cues therefore could not demonstrate a moderating effect of task-set dominance on attentional sensitization of priming in contrast to earlier work using color cues (Kiefer et al., 2019). This suggests that the moderating role of cue-task compatibility on task cue influences on masked semantic priming might depend on specific experimental settings.

## Supplementary information

ESM 1 (DOCX 59 kb)

## Footnotes

1 In fact, all stimuli were shown for a fixed number of frames according to the refresh rate of the screen. To facilitate comparison with earlier studies, we report the duration in milliseconds that correspond to the respective number of frames.

2 We would like to thank Iring Koch for providing this suggestion.

3 We would like to thank Thomas Kleinsorge for suggesting this analysis.

4 If the available trial number in one condition could not be divided by three, the second and/or third block consequently consisted of one trial less compared to the (second and) first block.

5 Note that it is unlikely that (partial) awareness of masked primes had influenced priming effects. Despite the fact that *d’* measures significantly deviated from zero, mean *d’* was small in both experiments and comparable to earlier studies (Kiefer, 2019; Kiefer et al., 2019; Kiefer & Martens, 2010; Ulrich et al., 2014). Furthermore, neither magnitude of RT nor ER priming correlated with *d’*. Hence, it is unlikely that awareness of prime content influenced the magnitude of priming, but differences in visual stimulation between letter strings and masked prime words could have biased responses in the prime identification task towards the correct response to a small degree.
