# Supplementary material for: Task cue influences on lexical decision performance and masked semantic priming effects: The role of cue-task compatibility
Source: Atten Percept Psychophys. 2022 Sep 20;84(8):2684–701. doi: 10.3758/s13414-022-02568-2 (PMC9630217; doi:10.3758/s13414-022-02568-2)
Supplement: Supplementary file 1 — (DOCX 59 kb) [file 13414_2022_2568_MOESM1_ESM.docx]

Supplementary Material

Task cue influences on lexical decision performance and masked semantic priming effects: The role of cue-task compatibility

**Berger, Kunde, & Kiefer**

**A: Sequence effects for experiment 1A and 1B**

In the present paradigm, prior to the primed lexical decision task (LDT), participants had to perform induction tasks associated with two different task sets (semantic vs. perceptual decision), which were signaled by task cues (induction task trials). Furthermore, there were trials, in which only the task cue without a following stimulus was presented (task cue-only trials). In task cue-only trials, only a response in the subsequent LDT was required. The sequence of trials was randomized. The preceding trial could thus include the same or a different task set and could be an induction task trial or a task cue-only trial, in which task sets were cued but not applied. As task set application (i.e. performing the cued task set) only occurred in induction task trials, sequence effects of task sets (the preceding trial included the same task set or not) and sequence effects of trial type (the preceding trial was an induction task trial or a task cue-only trial) are predominantly of interest for induction tasks. We therefore analyzed sequence effects for induction tasks in line with previous research combining induction task trials and task cue-only trials (Kiefer et al., 2019).

We conducted linear mixed model (LMM) analyses of response times (RT) and general linear mixed models (GLMM, using a binomial link function) of error rates (ER) in induction task.. LMM analyses were conducted using the package “lme4” (Bates et al., 2014) and “lmerTest” (Kuznetsova et al., 2017) for “R” (R Core Team, 2020) statistical software.

We calculated models including the task set and trial type repetition factors as well as all other factors included in the main analyses of induction tasks reported in the paper. All models included main effects as well as all possible interactions between the factors. We estimated linear mixed models with random intercepts per subject, if such models converged. All factors were contrast-coded. Preprocessing of data was identical as for the main text. As the inclusion of five experimental factors and all their interactions resulted in 32 effects (including the intercept), we will only report significant effects (*p* < 0.05) and effects involving the factors “type_rep” and “set_rep”.

Please note that these analyses should be considered exploratory, as the present study was not designed to investigate task switching / task repetition effects. For instance, trial sequences were entirely random and there was a self-paced break at the end of each trial, i.e. after the LDT.

Coding of factors:

type_rep: - ½ trial type switch ½ trial type repetition
set_rep: - ½ task set switch ½ task set repetition
set_c: - ½ perceptual task set ½ semantic task set
dom_c: - ½ weak task set dominance condition ½ dominant condition
exp_c: - ½ experiment 1A ½ experiment 1B

**Sequence effects in induction tasks for experiment 1A and 1B**

Table A1: Analysis of the sequence effects for response times in induction tasks collapsed across experiment 1A and 1B.

| Fixed effects | *beta* | *SE* | *t* | *p* |
| --- | --- | --- | --- | --- |
| type_rep*set_rep | -15.84 | 5.41 | -2.9 | 0.003 |
| type_rep*set_c*dom_c | -33.53 | 10.82 | -3.1 | 0.002 |
| type_rep*set_rep*set_c*exp_c | 50.29 | 21.61 | 2.3 | 0.020 |
| Random effects | *SD* |  |  |  |
| Intercept | 104.6 |  |  |  |

A significant interaction of the factors trial type repetition and task set repetition (type_rep*set_rep) was observed. RTs were especially slow if the trial type was switched but the task set was repeated. A trial type switch indicates that the preceding trial was a task cue-only trial, i.e. the task set was not applied in the previous trial. Elevated RTs in trial type switch and task set repetition trials could accordingly index inhibition of task sets in task cue-only trials. If a task set was cued but not applied in the preceding trial, re-activating the task set in order to perform a subsequent induction task is associated with RT costs, presumably reflecting demands for overcoming the previous inhibition of the task set (cf. Koch et al., 2010, 2018; Mayr & Keele, 2000). This effect was present in both experiments for both task sets, see *figure 1A* (left half of each panel). However, as indicated by the four-way interaction type_rep*set_rep*set_c*exp_c, for task set switches the pattern was less clear. Depending on task set and experiment, longer RTs were observed for trial type repetitions or switches, indicating cognitive reconfiguration processes to be more complex when the task set was switched. Furthermore, as indicated by the interaction type_rep*set_c*dom_c, the observed interaction (see the main text) of task set and task set dominance reflecting larger RT differences between task sets in the dominant task set condition, was further modulated by the preceding trial type. For trial type repetitions, induction task RTs were consistently faster for perceptual than semantic task sets (but more pronounced for dominant task sets). However, for trial type switches RTs for perceptual task sets were only faster for dominant task sets, while it was comparable across task sets in the weak task set condition. This could possibly reflect enhanced demands for implementing a task set in an induction task for weak task sets, as facilitatory influences of perceptual task sets diminished when participants had to switch from a task cue-only to an induction task trial.

Considering ERs, no effect involving the factors “set_rep” or “type_rep” showed a significant effect.


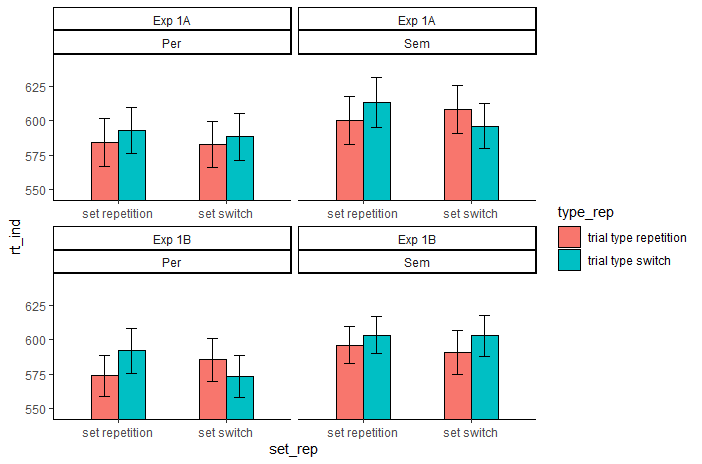


Figure A1: Response times in induction tasks depending on the factors type_rep, set_rep, task set and experiment. “Per” indicated a perceptual, “Sem” a semantic task set.

**B: Separate analyses for experiment 1A and 1B**

In the main text, we combined data of both experiment 1A and 1B for a joint statistical analysis. To control for possible influences, we included experiment as factor in these analyses. In this section, we will analyze effects, which were moderated by experiment, separately for each experiment (in the order as they appeared in the main text).

First, considering the analysis of RTs in the LDT, experiment and task set dominance further modulated the reported three-way interaction of task set, trial type and semantic relatedness. When performing the analyses separately, for experiment 1A the three-way interaction task set × trial type × semantic relatedness was significant, *F*(1, 49) = 9.01, *p* = 0.004, $\eta_{p}^{2}$ = 0.155, but not further significantly modulated by task set dominance: *F*(1, 49) = 2.96, *p* = 0.092, $\eta_{p}^{2}$ = 0.057. For experiment 1B, the three-way interaction was significant as well, *F*(1, 46) = 15.35, *p <* 0.001, $\eta_{p}^{2}$ = 0.250, but the additional modulation by task set dominance did not reach significance: *F*(1, 46) = 3.74, *p =* 0.059, $\eta_{p}^{2}$ = 0.075. Overall, the direction of the modulation was consistent across experiments and task set dominance conditions. However, when comparing the respective priming effect sizes (compare *table B1*), for dominant task sets, the modulation of priming was more pronounced in experiment 1B compared to 1A, while for weak task sets a stronger modulation was observed in experiment 1A compared to 1B. Overall, the modulation of priming by task sets and trial type was the weakest in the weak task set condition in experiment 1B, suggesting that the completely arbitrary task cues were most difficult to implement, resulting in a putatively lesser pronounced task set activation and therefore the smallest modulation of priming.

*Table B1:*Priming effect sizes for response times in the lexical decision task depending on experiment.

|  |  |  | ***d_priming*** | |
| --- | --- | --- | --- | --- |
| **Trial type** | **Task set dominance** | **Task set** | ***Experiment 1A*** | ***Experiment 1B*** |
| induction task trial | dominant | Per | 0.276 | 0.117 |
| induction task trial | dominant | Sem | 0.407 | 0.609 |
| task cue-only trial | dominant | Per | 0.437 | 0.419 |
| task cue-only trial | dominant | Sem | 0.362 | 0.243 |
| induction task trial | weak | Per | 0.209 | 0.232 |
| induction task trial | weak | Sem | 0.452 | 0.376 |
| task cue-only trial | weak | Per | 0.450 | 0.375 |
| task cue-only trial | weak | Sem | 0.189 | 0.326 |

Given is *Cohen's d* for the priming effects (see the section Statistical analyses in the main text).

Furthermore, the magnitude of priming depended on experiment, as indicated by the interaction semantic relatedness × experiment for ERs and drift rates. While ER priming was reliable and significant in both experiments (experiment 1A: *F*(1, 49) = 44.95, *p* < 0.001, $\eta_{p}^{2}$ = 0.478; experiment 1B: *F*(1, 46) = 102.69, *p <* 0.001, $\eta_{p}^{2}$ = 0.691), it was especially large in experiment 1B (*d_priming_* = -1.431) compared to experiment 1A (*d_priming_* = -0.745). Furthermore, drift rate priming was as well reliable in both experiments (experiment 1A: *BF* = 1.39*e^30^, experiment 1B: *BF* = 4.21*e^39^), but more pronounced in experiment 1B (*d_priming_* = 1.528) compared to 1A (*d_priming_* = 1.145). As the stimulus material and procedure (expect for the nature of the task cues) was identical across experiment 1A and 1B, it is difficult to explain this pattern. Possibly, different subject characteristics of the different samples of the two experiments resulted in elevated ER (and drift rate) priming in experiment 1B compared to 1A. However, as outlined above, the theoretical relevant modulation of priming by task set and trial type could be observed in both experiment, and therefore appears to be unaffected by the different magnitude of (ER / drift rate) priming across experiments.

Lastly, RTs in induction tasks depending on block number differed according to the experiment, indicated by the four-way interaction task set × task set dominance × block × experiment. Separately calculated per experiment, the interaction task set × task set dominance × block neither reached significance for experiment 1A (*F*(1, 49) = 3.48, *p* = 0.068, $\eta_{p}^{2}$ = 0.066) nor for experiment 1B (*F*(1, 46) = 1.92, *p* = 0.173, $\eta_{p}^{2}$ = 0.040). As can be seen in *table B2*, induction task RTs for perceptual task sets were consistently faster compared to semantic task sets despite for the 1^st^ block for weak task sets in experiment 1B. Implementation and accordingly applying task sets was presumably the most difficult in the weak task set condition in experiment 1B, where task cues were completely arbitrary and task cues and tasks therefore lacked any compatibility. Hence, in the 1^st^ experimental block, when task set application in induction tasks was only practiced for a short duration, differences in induction task RTs across task sets may have been concealed, as performance did not yet reach a level where any influences of task set difficulty could be revealed.

*Table B2:*Effect sizes for the comparison perceptual - semantic task set for response times in induction tasks.

|  |  | ***d(Per - Sem)*** | |
| --- | --- | --- | --- |
| **Block** | **Task set dominance** | ***Experiment 1A*** | ***Experiment 1B*** |
| 1st | dominant | -0.149 | -0.351 |
| 3rd | dominant | -0.248 | -0.493 |
| 1st | weak | -0.174 | 0.169 |
| 3rd | weak | -0.005 | -0.093 |

Effect sizes were calculated by subtracting the response time of semantic minus the response time of perceptual task sets in the respective condition and dividing through the mean SD of both task sets in the respective condition.

**C: Descriptive statistics of the N400 ERP component**

*Table C1:*
Descriptive statistics of the N400 component for experiment 1A.

|  |  |  |  |  | **left hemisphere** | | **right hemisphere** | |
| --- | --- | --- | --- | --- | --- | --- | --- | --- |
| **Task set dominance** | **Task set** | **Trial type** | **Semantic relatedness** |  | ***Mean*** | ***SD*** | ***Mean*** | ***SD*** |
| dominant | semantic | induction task trials | related |  | 0.28 | 3.00 | 1.19 | 2.97 |
|  |  |  | unrelated |  | -0.64 | 2.71 | 0.69 | 2.93 |
|  |  | task cue-only trials | related |  | 0.86 | 3.80 | 1.92 | 3.85 |
|  |  |  | unrelated |  | -0.01 | 3.39 | 0.97 | 3.21 |
|  | perceptual | induction task trials | related |  | 0.78 | 3.13 | 1.86 | 3.66 |
|  |  |  | unrelated |  | 0.26 | 3.22 | 1.40 | 3.51 |
|  |  | task cue-only trials | related |  | 1.10 | 3.74 | 2.32 | 3.37 |
|  |  |  | unrelated |  | 0.10 | 3.25 | 1.42 | 3.12 |
| weak | semantic | induction task trials | related |  | 0.84 | 3.75 | 1.27 | 2.86 |
|  |  |  | unrelated |  | -0.42 | 3.34 | 0.05 | 2.53 |
|  |  | task cue-only trials | related |  | 0.56 | 3.22 | 1.04 | 2.84 |
|  |  |  | unrelated |  | -0.34 | 3.61 | 0.29 | 3.52 |
|  | perceptual | induction task trials | related |  | 1.49 | 3.40 | 2.02 | 2.95 |
|  |  |  | unrelated |  | 0.38 | 3.57 | 1.16 | 3.01 |
|  |  | task cue-only trials | related |  | 0.89 | 3.32 | 1.63 | 3.33 |
|  |  |  | unrelated |  | 0.03 | 3.42 | 1.02 | 3.47 |

The unit is µV. Values for a hemisphere were calculated by averaging the activity of the respective electrodes in this hemisphere.

*Table C2:*
Descriptive statistics of the N400 component for experiment 1B.

|  |  |  |  | **left hemisphere** | | **right hemisphere** | |
| --- | --- | --- | --- | --- | --- | --- | --- |
| **Task set dominance** | **Task set** | **Trial type** | **Semantic relatedness** | ***Mean*** | ***SD*** | ***Mean*** | ***SD*** |
| dominant | semantic | induction task trials | related | 0.18 | 3.33 | 1.17 | 3.02 |
|  |  |  | unrelated | -1.13 | 3.27 | 0.20 | 2.93 |
|  |  | task cue-only trials | related | 2.71 | 3.64 | 3.20 | 3.29 |
|  |  |  | unrelated | 1.76 | 3.36 | 2.51 | 3.04 |
|  | perceptual | induction task trials | related | 0.80 | 3.37 | 1.77 | 3.28 |
|  |  |  | unrelated | 0.00 | 2.87 | 1.13 | 2.70 |
|  |  | task cue-only trials | related | 2.05 | 3.46 | 3.04 | 3.18 |
|  |  |  | unrelated | 1.42 | 2.92 | 2.61 | 2.68 |
| weak | semantic | induction task trials | related | -0.78 | 3.24 | 0.61 | 2.67 |
|  |  |  | unrelated | -1.58 | 2.89 | -0.08 | 2.52 |
|  |  | task cue-only trials | related | 0.59 | 2.58 | 1.20 | 2.61 |
|  |  |  | unrelated | -0.28 | 2.29 | 0.44 | 2.41 |
|  | perceptual | induction task trials | related | -0.23 | 3.05 | 0.97 | 2.87 |
|  |  |  | unrelated | -0.70 | 3.28 | 0.68 | 3.04 |
|  |  | task cue-only trials | related | 0.64 | 2.69 | 1.35 | 2.69 |
|  |  |  | unrelated | 0.05 | 2.71 | 1.00 | 2.82 |

The unit is µV. Values for a hemisphere were calculated by averaging the activity of the respective electrodes in this hemisphere.

Supplementary references

Bates, D., Mächler, M., Bolker, B. M., & Walker, S. C. (2014). Fitting Linear Mixed-Effects Models using lme4. *Journal of Statistical Software*, *67*(1). https://doi.org/10.18637/jss.v067.i01

Kiefer, M., Trumpp, N. M., Schaitz, C., Reuss, H., & Kunde, W. (2019). Attentional modulation of masked semantic priming by visible and masked task cues. *Cognition*, *187*, 62–77. https://doi.org/10.1016/j.cognition.2019.02.013

Koch, I., Gade, M., Schuch, S., & Philipp, A. M. (2010). The role of inhibition in task switching: A review. *Psychonomic Bulletin and Review*, *17*(1), 1–14. https://doi.org/10.3758/PBR.17.1.1

Koch, I., Poljac, E., Müller, H., & Kiesel, A. (2018). Cognitive structure, flexibility, and plasticity in human multitasking-an integrative review of dual-task and task-switching research. *Psychological Bulletin*, *144*(6), 557–583. https://doi.org/10.1037/bul0000144

Kuznetsova, A., Brockhoff, P. B., & Christensen, R. H. B. (2017). lmerTest Package: Tests in Linear Mixed Effects Models. *Journal of Statistical Software*, *82*(13), 1–26. https://doi.org/10.18637/JSS.V082.I13

Mayr, U., & Keele, S. W. (2000). Changing internal constraints on action: The role of backward inhibition. *Journal of Experimental Psychology: General*, *129*(1), 4–26. https://doi.org/10.1037/0096-3445.129.1.4

R Core Team. (2020). *R: A Language and Environment for Statistical Computing*.
